# Advances on Cellulose Manufacture in Biphasic Reaction Media

**DOI:** 10.3390/ijms241512404

**Published:** 2023-08-03

**Authors:** Marcos Fernández-Bautista, Sergio Martínez-Gómez, Sandra Rivas, José Luis Alonso, Juan Carlos Parajó

**Affiliations:** 1 Faculty of Science, Chemical Engineering Department, University of Vigo (Campus Ourense), Polytechnical Building, As Lagoas, 32004 Ourense, Spain; marcosinfor10@gmail.com (M.F.-B.); sergiomg691@gmail.com (S.M.-G.); sandrarivas@uvigo.es (S.R.); xluis@uvigo.es (J.L.A.); 2 CINBIO, University of Vigo (Campus Lagoas-Marcosende), 36310 Vigo, Spain

**Keywords:** cellulose, lignocellulose, fractionation, biphasic media

## Abstract

Cellulose is produced industrially by the kraft and sulfite processes. The evolution of these technologies in biorefineries is driven by the need to obtain greater added value through the efficient use of raw materials and energy. In this field, organosolv technologies (and within them, those using liquid phases made up of water and one partly miscible organic solvent, known as “biphasic fractionation” in reference to the number of liquid phases) represent an alternative that is receiving increasing interest. This study considers basic aspects of the composition of lignocellulosic materials, describes the fundamentals of industrial cellulose pulp production processes, introduces the organosolv methods, and comprehensively reviews published results on organosolv fractionation based on the use of media containing water and an immiscible solvent (1-butanol, 1-pentanol or 2-methyltetrahydrofuran). Special attention is devoted to aspects related to cellulose recovery and fractionation selectivity, measured through the amount and composition of the treated solids.

## 1. Lignocellulosic Biomass (LB) as a Resource for the Sustainable Development

The current socio-economic development model, which relies heavily on the consumption of increasingly scarce fossil resources, has reached a crisis point. Some important contributing factors are the population growth, the fact that oil and gas are geographically concentrated (jeopardizing the security of supply), the uncertainty caused by price volatility, and environmental issues (particularly those related to greenhouse gas emissions and consequent global warming).

The substitution of fossil resources with renewable ones would contribute to solving these problems, and could have a positive economic and social impact on the primary sector (e.g., through the exploitation of set-aside land). This is one of the principles of sustainable development, which aims to meet the current needs of the population without compromising the resources and possibilities of future generations [1].

Vegetal biomass (VB) is a renewable and sustainable resource that represents the most abundant organic carbon source on Earth. There are many types of VB, which may have very different chemical compositions. Some types of VB, such as cereal grains, are used as food, and their large-scale industrial use for other purposes (i.e., bioethanol manufacture) would limit the amount available for use as food or feed. In contrast, other types of plant biomass (such as those containing high proportions of cellulose, commonly referred to as cellulosic materials, lignocellulosic materials or lignocellulose) do not have food applications, are generated in large quantities (equivalent to 80% of the total plant biomass) [2,3], and are suitable for processing into products of commercial interest (see below). In this field, Giuliano [4] reported on the transition to a new scenario defined by the manufacture of biofuels and biochemicals from biomass, whereas Wang et al. [5] highlighted the negative effects of using feedstocks suitable as foods (e.g., corn starch) as raw materials for industry, which could drive a surge in prices and endanger food security. 

The VB of a lignocellulosic nature is mainly made up of a polyphenolic fraction called lignin, and polysaccharides, including cellulose and hemicelluloses. Forests are the major source of lignocellulosic biomass (LB), which is also produced in grasslands or crops, or corresponds to agricultural byproducts such as straws or stalks. The total aboveground biomass stored in EU forests reached 18,600 Mt in 2013, and the average annual harvest accounts for 63% of the growth rate [6].

LB shows enormous potential as a feedstock for environmentally friendly and sustainable chemical processes, due to its huge and widespread availability, and its low price. The resulting bioproducts are expected to contribute to a new bioeconomy through efficient energy use and waste minimization, with reduction in the carbon footprint, together with the reuse of byproducts and wastes, in accordance with the principles of the “circular economy”.

## 2. LB Composition

The main factor hindering the use of lignocellulosic materials as industrial feedstocks is their complex and heterogeneous nature. LB is made up of non-structural components (such as resins, inorganic components, or proteins) and structural components, which include two types of polysaccharides (cellulose and hemicelluloses), and a polymer of a phenolic nature (lignin). Typically, the added value that can be obtained from LB is concentrated in the structural components, whose major chemical features are as follows:

- Cellulose is a long-chain, unbranched polymer made up of anhydroglucose units linked together by β-1,4-glycosidic bonds, in the form of cellobiose subunits, forming long chains that are bundled into microfibrils.
- Hemicelluloses are structurally diverse, branched heteropolysaccharides, whose chains can be made up of diverse anhydrosugars (including anhydroxylose, anhydroarabinose, anhydromannose, anhydrogalactose, and anhydroglucose), linked together by different types of glycosidic bonds, and that may be substituted with acetyl or *O*-methyluronic groups. The type and proportions of anhydrosugars depend on the type of LB considered.
- Lignin is an amorphous, hydrophobic, cross-linked, three-dimensional, branched polymer made up of three phenylpropanoid structural units (*p*-hydroxyphenyl, denoted as H, derived from *p*-coumaryl alcohol; guaiacyl, denoted as G, derived from coniferyl alcohol; and syringyl, denoted as S, derived from sinapyl alcohol). These monomers are linked together through a diversity of inter-unit linkages such as C-C (β-β′, β-5′, β-1′, and 5-5′) and ether bonds (β-*O*-4′, α-*O*-4′, and 5-*O*-4′), formed during lignin biosynthesis by the action of laccase and peroxidase enzymes [7,8].

Although the composition of the diverse LB types shows wide variation ranges, typical feedstocks contain 35–50% cellulose, 15–30% hemicelluloses, and 20–30% lignin, with 80–90% of the feedstock dry weight corresponding to the joint contribution of the structural components.

**Figure 1:**
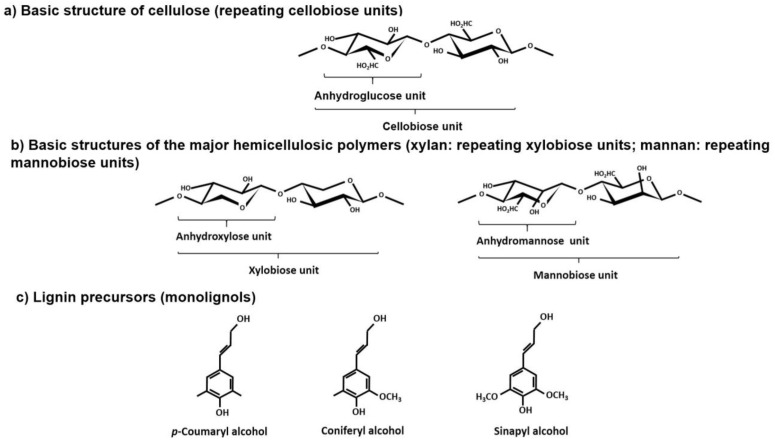
Chemical structures of: (**a**) cellobiose, a constituent of cellulose; (**b**) xylobiose and mannobiose, constituents of the major hemicellulosic polymers (xylan and mannan); (**c**) lignin precursors.

Figure 1 shows the major structural units of cellulose and hemicellulosic polysaccharides, as well as the precursors of lignin.

In plant cell walls, cellulose, hemicelluloses and lignin are intertwined and closely interconnected, providing strength and rigidity, resulting in a complex and highly organized network. Lignin fills the void spaces in this matrix, strengthens the cell wall, and increases the resistance to chemical and biological degradation.

## 3. Chemical Processes for Cellulose Manufacture

The industrial production of cellulose pulp requires the deconstruction of the lignocellulose matrix by chemical processing, which is based on the different properties of the constituent polymers. Thus, cellulose is more resistant to the action of alkalis than hemicelluloses, which are also more susceptible to hydrolysis by acids, while lignin can be solubilized in aqueous media containing inorganic pulping chemicals or via the action of selected organic solvents. In pulping industries, the chemical processing of the raw materials is oriented towards the solubilization of lignin, leaving cellulose in the solid phase. Hemicelluloses are solubilized to an extent that depends on the technology used.

Currently, about 90–91% of the chemical pulp is produced from wood [9,10], which presents advantages over other potential raw materials in terms of the abundance of supply, year-round availability, and high cellulose content. In developing countries, low-cost raw materials are used as alternative resources for pulp and paper production, due to the cost and limited availability of wood. In these countries, about 60% of cellulose comes from non-wood LB, including agricultural by-products of cereal cultivation, industrial crops (such as hemp, sugarcane and kenaf), and naturally grown crops (such as bamboo, reeds and grasses). The most commonly used non-woody raw materials are straws, followed by bagasse, reeds, and bamboo [10].

### 3.1. Commercial Processes

**Figure 2:**
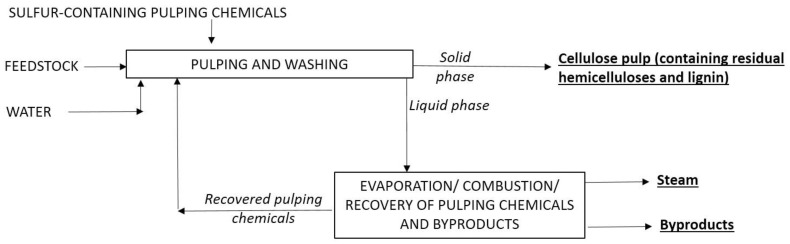
Operational principle of the commercial pulping technologies.

At the industrial level, the chemical pulp production is dominated by the kraft and sulfite processes, which use sulfur-containing chemicals to remove most of the lignin and hemicelluloses from the raw material, leaving a solid phase composed mainly of cellulose, which also contains minor amounts of residual hemicelluloses and lignin. The general idea of the commercial processes for pulp manufacture is shown in Figure 2. In 2021, the world production of chemical pulp accounted for 151 × 10^6^ metric tons [11].

The kraft pulp, which accounts for approximately 80% of all chemical pulp produced worldwide, uses aqueous solutions of sodium sulfide and sodium hydroxide to solubilize most of the lignin and a portion of the hemicelluloses, operating at 150–180 °C for about 2 h. The commercial success of this process is based on a number of factors, including the favorable pulp yield and excellent mechanical properties of the pulp, while the surplus steam is usually employed to generate green electricity which is used in the pulp mill or sold to the public power grid. 

The sulfite process is the oldest method of obtaining pulp from wood, which has been largely superseded by the kraft process (which is more efficient and produces pulps with better mechanical properties), but remains competitive for specific applications. The production of chemical pulp is performed by treating the raw material with solutions containing sulfurous acid (obtained by dissolving SO_2_ in water) in the presence of other chemicals employed to modify the pH of the pulping liquor.

The kraft and sulfite pulps are brownish and contain residual lignin and hemicelluloses. Depending on the specific requirements of the end application, higher-quality pulps (brighter and/or with higher cellulose content) may be required. This can be achieved by means of further multistage processing (bleaching sequences) performed with a number of chemicals, including gases (oxygen, ozone), alkalis, hydrogen peroxide, chlorine and/or chlorine-containing chemicals. The sequence of bleaching stages to be employed in each case depends largely on the properties of the raw pulps, as well as on the applications for which they are intended. 

### 3.2. Evolution of Pulp Mills to LB Biorefineries

Figure 2 shows that the commercial pulp mills mainly produce pulp and energy from a lignocellulosic feedstock. A generalization of this idea is crystallized in the LB biorefinery concept, which proposes the integral conversion of LB into a range of value-added products, including biofuels, biochemicals, and biomaterials. The biorefinery concept is analogous to that underlying the current petroleum refineries, which produce multiple fuels and products from oil. In LB biorefineries, the feedstock is subjected to chemical “fractionation” resulting in separate streams that contain the major LB components (or products derived from them), allowing the individual utilization of cellulose, hemicelluloses and lignin. In subsequent processing stages, the fractionation streams can be processed into a range of market products, with minimal waste production. This approach extracts the maximum added value from the feedstock, and represents a valuable alternative for the sustainable production of a wide range of products from LB, facilitating the transition towards a more circular and sustainable economy.

The pulp industry strives to improve its profitability by implementing the above philosophy. For example, in the kraft process, a number of byproducts (such as tall oil or turpentine) can contribute to the economics. In turn, using kraft lignin to produce high-value-added products is a major challenge, due to its complex structure, poor reactivity, and low solubility, which are factors that limit the lignin’s large-scale use for chemical purposes [12]. However, lignin fractions recovered from the kraft pulping media present potential as substrates for thermochemical conversion, including pyrolysis, gasification, and hydrothermal liquefaction), or as starting materials for the manufacture of adhesives, resins, carbon fibers, adsorbents, dispersants, emulsifiers, or binding agents [12,13,14,15,16,17].

In the sulfite mills, the hemicellulose-derived products present in the reaction media can be processed and refined to recover acetic acid, xylose (which can be converted in xylitol or fermented into ethanol) and lignosulfonates [18,19,20,21,22]. The latter can be used as concrete additives, soil stabilizers, dust suppressants, deflocculants to reduce the viscosity of drilling mud, and animal feed [18,23,24,25].

However, the commercial technologies for pulp production present some drawbacks, mainly related to the use of products derived from hemicelluloses and lignin. Hemicelluloses are burned in the kraft process, which is a low-added-value application for a complex biosynthetic polymer, while the recovery of hemicellulose hydrolysis products in the sulfite mills is complex and costly. Lignins from both commercial processes contain sulfur (which is incorporated into the lignin fragments as thiol groups in kraft lignin, or as sulfonic groups in sulfite lignin), making their chemical utilization difficult (e.g., due to their negative effects on metal catalysts) [26]. In addition, the manufacture of higher-added-value pulps (for example, dissolving pulps employed to make cellulose derivatives) requires modifications of the kraft process (by implementing a hemicellulose removal step, in the process known as prehydrolysis-kraft) or costly treatments of sulfite pulps with concentrated alkali to remove residual hemicelluloses.

### 3.3. Organosolv Fractionation of LB

An ideal LB fractionation technology should improve the drawbacks of the commercial process, leading to biorefineries capable of generating individual products from side streams, which would provide great opportunities for climate protection, value creation, and resource efficiency in the context of the bioeconomy [18]. In particular, organosolv technologies are among the most promising strategies for LB valorization, and could facilitate the transition to an enhanced utilization of renewable feedstocks [27].

Organosolv fractionation, based on the use of organic solvents that are feasible to recover [28], is considered a promising alternative for LB processing [27,29,30,31,32]. 

Most organosolv pretreatments are primarily based on delignification, which proceeds through the formation of low-molecular-weight lignin fragments that dissolve in the reaction media [7].

The type of solvent to be employed in a specific process depends on the considered feedstock, and defines the resulting fractionation products. The solvent properties (including boiling point, green character, water solubility, ease of recovery, and delignification efficiency) are key determinants for a proper choice [30]. The organosolvents employed for LB fractionation include alcohols (from methanol to pentanol), polyols (glycerol, ethylene glycol, propylene glycol), short-chain organic acids (formic, acetic), ketones (acetone, methyl-isobutyl ketone), γ-valerolactone (GVL), and furanic compounds (tetrahydrofuran, methyltetrahydrofuran) [30,33,34,35,36,37]. In general, non-toxic solvents obtainable from renewable resources are preferred, as they allow a more sustainable operation.

Typically, organosolv fractionation media consist of water and the selected solvent, often together with a catalyst [30]. Although a number of catalysts have been considered in the literature, most studies use acid-catalyzed media, in which the increased hydronium ion concentrations allow an effective fractionation under mild operational conditions. Organosolv processes are versatile (in terms feedstock types or commercial processes) and can be performed under mild conditions to achieve a selective separation of fractions with minimal waste production.

The main fractionation effects caused by organosolvents can be summarized as follows:

- Lignin is selectively solubilized by means of the breakage of the α-*O*-aryl and β-*O*-aryl bonds in lignin [30,36], and the lignin fragments remain dissolved in the organic solvent [34], from which they are recovered by adding an antisolvent (typically, water or acidic solutions) that causes their precipitation [29,35,37,38]. The recovered sulfur-free lignin fractions are of superior quality [30], showing improved properties for further chemical utilization.
- Depending on the presence of catalysts and operational conditions, hemicelluloses can be partly retained in the solid phase [34], or extensively solubilized and converted into a variety of hydrolysis or hydrolysis-dehydration products (low molecular weight polymers, oligosaccharides, monosaccharides or furans). After lignin precipitation, hemicellulose-derived products remain in the liquid phase, along with acid soluble lignin, carbohydrate degradation products, organic acids, and other components [7]. The recovery of saccharides from the media is achieved in subsequent processing stages.
- Depending on the process considered, cellulose can be recovered at high yield, or converted into valuable chemicals (oligosaccharides, glucose, 5-hydroxymethylfurfural, or stoichiometric mixtures of levulinic and formic acids) in one-pot operations. This article is focused on fractionation processes in which cellulose is kept in the solid phase. Upon organosolv processing, cellulose may undergo chemical and structural changes (reduction in polymerization degree and/or crystallinity) that increase its susceptibility to hydrolysis by acids or enzymes, facilitating applications based on fermentation (such as biofuels or biochemical manufacturing).

**Figure 3:**
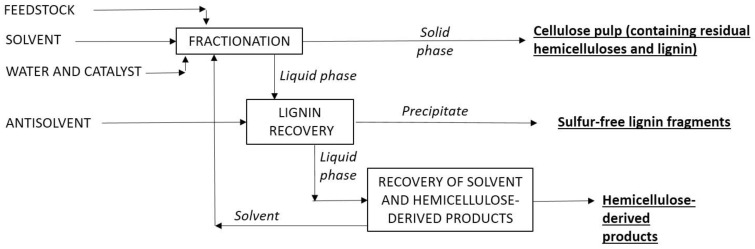
Operational principle of conventional organosolv fractionation processes.

Figure 3 shows the general principle of organosolv fractionation. It can be noted that this operational mode enables the separation of the three main LB components, and their further individual utilization for specific purposes. An important aspect is the facilitated solvent recycling, product recovery and product purification, which are crucial aspects defining the overall profitability of the process [27]. Volatile solvents can be recovered via evaporation, whereas more complicated schemes are needed for non-volatile solvents. However, important challenges remain to be resolved for the widespread implementation of organosolv biorefineries, particularly those related to profitability, energy demand, solvent type and recycling, robustness regarding biomass type and the integration of hemicellulose recovery and use [34].

## 4. Processes for Cellulose Manufacture Based on Reaction Media Made up of Water and One Partially Miscible Solvent

### 4.1. Principles of Operation

**Figure 4:**
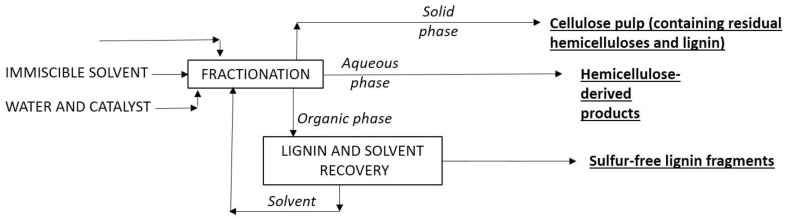
Operational principle of FMCWPMS processes.

Increasing attention is being paid to the organosolv fractionation of LB using solvents of low water solubility at room temperature such as 1-butanol (BuOH), 1-pentanol (PenOH), or 2-methyltetrahydrofuran (MeTHF). When LB is processed in reaction media consisting of water, the partially miscible solvent, and (optionally) a catalyst, fractionation provides a cellulose-enriched solid, an aqueous phase containing the polar compounds (accumulating the hemicellulose-derived products, including saccharides or saccharide-derived products such as furans and organic acids), and a solvent-rich phase that accumulates the soluble lignin fragments [29,31,37,38]. Based on the number of liquid phases, this operational method is known in the literature as “biphasic fractionation”, and is denoted here with the acronym FMCWPMS (fractionation in media containing water and one partly miscible solvent). Figure 4 shows the general idea of FMCWPMS methods.

With this approach, the three LB constituent polymers are fractionated in a single reaction stage (“one-pot” operation) and spontaneously separated in different streams, defining an efficient framework for the sustainable manufacture of a scope of bioproducts. These technologies can follow the principles of green chemistry and cleaner production, and offer a potential solution for the creation of an economically viable biomass upgrading process [39]. In economic terms, the FMCWPMS is expected to reduce the costs of separation and purification of the target products, as well as those related to solvent recovery and reuse. Moreover, these methods enable efficient lignin separation, which is important from a biorefinery perspective since lignin valorization is crucial to develop economically feasible strategies for LB utilization [40].

### 4.2. BuOH-Based Fractionation

BuOH is one of the most important solvents for FMCWPMS due to multiple reasons, including the possibility of being manufactured from renewable raw materials, its environmentally friendly nature, and its ability to dissolve high-quality lignin fragments [41]. Although BuOH and water are miscible at high temperatures, they become partially miscible at room temperature, at which the reaction media yield organic and aqueous layers. Phase separation takes place with BuOH volume percentages as low as 12.5% [42].

Diverse LB types have been processed in BuOH–water media, including highly lignified materials [29,31,43,44,45], agricultural products and byproducts [29,31,42,43,45,46,47,48,49,50,51,52,53,54], and dedicated energy crops [41]. However, some of these studies are focused on topics such as lignin isolation and characterization or enzymatic hydrolysis, precluding the discussion of aspects related to the overall phase separation and selectivity.

The relevant information for the purposes of this study is summarized in Table 1. Several facts make the interpretation of results difficult: for example, the studies deal with different raw materials, which may show large compositional differences; the operational conditions present large variation ranges (in particular, temperature, reaction time, relative amount of solid, proportion of BuOH in the medium, and type and concentration of catalyst, if used); various types of reactors have been employed (batch with conventional heating, microwave-heated batch, flow-through); and the objectives are also diverse (fractionation focused on exhaustive removal of hemicelluloses and lignin, fractionation aiming at the selective separation of compounds with limited cellulose losses; isolation of high-quality lignins, manufacture of solids susceptible to enzymatic hydrolysis, etc.). On the other hand, the values of key variables measuring the selectivity of component separation (solid recovery yield and/or composition of treated solids) is missing in some studies (particularly those ones dealing with lignin recovery from fractionation media or cellulose saccharification).

It is observed that the composition of raw materials is very influential on fractionation. In general, non-woody biomass is easier to pretreat than woody biomass [55]. Softwoods (such as Japanese cedar, Douglas fir wood or pine wood) show poor susceptibility to BuOH fractionation in uncatalyzed media [29] or when Brønsted or Lewis acids are employed as catalysts, resulting in treated solids with comparatively high lignin contents (26.3–16.93%) [43,44,45], with significant cellulose losses [31]. When processing softwoods, lignin removal can be improved using NaOH as a catalyst [44], which shows advantages with respect to minerals or weak acids [56]. The limited susceptibility of softwoods to delignification treatments has been justified both by their higher lignin contents and by structural features related to the relative proportions of the various lignin structural units [31]. In comparison, hardwoods (eucalypt and beech) were efficiently fractionated in media catalyzed with H_2_SO_4_. In particular, excellent results (treated solids containing 86.3% cellulose, 1.8% lignin and 1.4% hemicellulose) were obtained using a flowthrough reactor [45]. Less lignified materials (such as herbs, bagasses, straws, husks, shells, reeds, and industrial byproducts) were more susceptible to BuOH fractionation, although the information on this phenomenon is scarce and incomplete. Based on the data reported by Ghose et al. [48], it is clear that the operational conditions must be optimized for each raw material: for example, under the same experimental conditions, the delignification percentage achieved for bagasse (88.1) was much higher than that obtained with jute sticks (36.2). Cellulose contents above 83% have been reported for solids coming from the fractionation of raw or water-extracted *Arundo donax* [41] and walnut shells [45].

Although a deep discussion on fractionation’s effects should take into account both the degree of delignification and cellulose losses, this information is provided in just a few articles. For example, Ghose et al. [48] reported 36.2–88.1% delignification when the raw materials considered were treated under fixed operational conditions, but the composition of the feedstocks was missing, which prevented the calculation of cellulose losses. Schmetz et al. [31], operating with a limited BuOH percentage (33%) at fixed values of temperature, catalyst concentration and reaction time, obtained good results with hardwoods and other susceptible substrates (72–87% delignification with 15–36% cellulose loss), suggesting that the results could be improved with individual optimization for the various raw materials. In an optimization study, Rivas et al. [41] reported up to 86.7% delignification of *Arundo donax*, with cellulose losses in the range of 12.1–33.3%. Comparatively better results (up to 85.4% delignification with 9.96–15.4% cellulose loss) were reported in the same article for assays performed with water-extracted *Arundo donax*. Related results (62–74% delignification with 9.8–37.7% cellulose loss) were obtained by Salapa et al. [49] when wheat straw was treated in media containing 50% BuOH. Treatments or sorghum bagasse in media containing less BuOH (12.5%) did not improve the delignification extent (64.7%), but resulted in better selectivity, with limited cellulose loss (8.9%) [42]. In a further optimization study, in which the BuOH content of media was considered as an operational variable, the same authors achieved higher degrees of delignification (up to 76%) with very good selectivity (2–8% cellulose loss) [51].

Based on the above results, it can be inferred that treatments in media with moderate–low BuOH contents enable a satisfactory degree of delignification, but can lead to significant cellulose solubilization, an aspect directly related to the limited solid yields cited in the literature [50]. On the contrary, operation in BuOH-rich media improves cellulose recovery.

The interest of BuOH fractionation also stems from the chemical modifications undergone by hemicelluloses and lignin. Operating in media with high BuOH proportions, the hemicellulose hydrolysis can proceed (at least in part) with the simultaneous incorporation of BuOH in the anomeric position the generated monosaccharides. The butoxylated monosaccharides can be employed for specific purposes (e.g., as valuable synthetic building blocks) [43], or subjected to further chemical and/or enzymatic reactions to yield the corresponding sugars [54]. 

From a biorefinery perspective, the utilization of lignins is of paramount importance. One of the advantages of BuOH fractionation is that the isolated lignins show low contamination by polysaccharides [31]. When the reaction is performed in media containing high BuOH concentrations and an acidic catalyst, the alcohol is incorporated into the β-aryl ether structure via α-alkoxylation [45], facilitating the extraction of lignin and preventing the formation of C–C bonds via condensation reactions [52]. For these reasons, these lignins show favorable characteristics to be employed as substrates for the manufacture of aromatic chemicals [43] or as precursors of new materials [54]. When fractionation is carried out under harsh conditions, the lignins may react with sugar-dehydration products (furans) to yield a new type of compound (“pseudolignins”) with specific properties [47].

**Table 1:** Results reported on the BuOH fractionation of lignocellulosic materials.

Substrate	BuOH:Water Ratio; Catalyst (Conc.)	Temp.; Liquor to Solid Ratio; Time	Solid Yield	Cellulose, %	Klason Lignin, %	Hemicellulose, %	Delignification, %	Cellulose Loss, %	Reference
Japanese cedar wood	1:1–1:49 mol/mol; uncatalyzed	200 °C; n.r. 120 min	n.r.	n.r.	n.r.	n.r.	27–53	n.r.	[29]
Willow wood	1:4 mol/mol; uncatalyzed		n.r.	n.r.	n.r.	n.r.	86	n.r.	
Sugarcane bagase			n.r.	n.r.	n.r.	n.r.	67	n.r.	
Japanese cedar wood	1:3 *v*:*v*; H_2_SO_4_ (1%)	180 °C; 13.3 mL/g; 45 min	n.r.	n.r.	n.r.	n.r.	12	22	[31]
*Eucalyptus* wood							78	20	
Beech wood							72	18	
Sugar cane bagasse							87	15	
Sugarbeet pulp							82	36	
Tall fescue							87	18	
Beech wood	95:5 *w*:*w*; HCl (0.2 M)	NBT (reflux); - ;360 min	46	n.r.	21	n.r.	n.r.	n.r.	[43]
Douglas fir wood			57	n.r.	17	n.r.	n.r.	n.r.	
Walnut shell			35	n.r.	32	n.r.	n.r.	n.r.	
*Pinus contorta* wood	78:22 *w*/*w*; MgCl_2_ (0.025 M)	200 °C; 100 g/L; 60 min	46.2	75.02	18.02	2.01	n.r.	n.r.	[44]
		205 °C; 100 g/L; 30 min	47.4	77.30	17.3	2.44	n.r.	n.r.	
	65:35 *w*/*w*; H_2_SO_4_ (1.1%)	170 °C; 100 g/L; 60 min	45.0	74.64	18.01	2.35	n.r.	n.r.	
			44.5	77.30	16.93	1.6	n.r.	n.r.	
	65:35 *w*/*w*; NaOH (2%)		44.6	70.01	6.94	12.6	n.r.	n.r.	
Rice straw	1:1 *v*:*v*; aromatic acid (0.5%)	120 °C; 12.5 mL/g; 120 min	54.2	n.r.	n.r.	n.r.	82.8	n.r.	[48]
Bagasse			44.0				88.1		
Rice husks			57.1				69.3		
Banana stalks			59.4				62.3		
Wheat straw			44.8				79.4		
Jute sticks			61.3				36.2		
Wheat straw	1:1 *v*:*v*; H_2_SO_4_ (0.023 M)	160–180 °C; 20 mL/g; 20–40 min	n.r.	67.1–71.0	17.9–23.2	0.4–3.3	62-74	9.8–37.7	[49]
*Arundo donax*	33:67 *v*/*v*; H_2_SO_4_ (1–3%)	150–190 °C; 15 mL/g; 20 min	28.7–75.5	40.2–83.7	7.2–20.3	1.7–24.5	17.1-86.7	12.1–33.3	[41]
*Arundo donax* (water extracted)	23:77–43/57 *v*/*v*; H_2_SO_4_ (0.5–1.25%)	155–170 °C; 15 mL/g; 15–20 min	38.8–83.0	42.0–84.3	8.8–22.7	4.2–26.1	19.5–85.4	9.9–15.4	
Sorghum bagasse	10:70 *v*/*v*; H_2_SO_4_ (1%)	180 °C; 13.3 mL/g; 45 min	48	59.1	32	3	64.7	8.9	[42]
Sorghum bagasse	6.25:93.75-37.5:62.5 *v*/*v*; H_2_SO_4_ (1%)	180 °C; 13.3 mL/g; 45 min	39.3–56.1	52.3–73.8	11–29	4.8–5.7	63.6–76.0	2–8	[51]
Walnut shells	240:78 *v*/*v*; HCl (2.16 M)	120 °C; 10.6 mL/g; 24 h	n.r.	n.r.	n.r.	n.r.	50	n.r.	[52]
Walnut shells	9:1 *v*/*v*; H_2_SO_4_ (0.1–0.18 M)	120 °C; 15 mL flowthrough/g; 2.5 h	n.r.	83.6	8.4	7.3	85	n.r.	[45]
Beech wood				86.3	1.8	1.4	n.r.		
Reed				71.4	2.3	2.2			
Douglas fir wood				73.0	26.3	6.2			
Pine wood				42.9	n.r.	1.0			
Vine shoots	30:70 *v*/*v*; H_2_SO_4_ (0–2%)	160–190 °C; 12 mL/g; 20 min	39.5–43.5	n.r.	n.r.	n.r.	24.7–50.0	n.r.	[53]
Vine shoots (water extracted)	30:70–60:40 *v*/*v*; H_2_SO_4_ (0–2%)	160–190 °C; 12 mL/g; 20 min	39.3–72.0	41.9–75.9	n.r.	n.r.	0-63.5	n.r.	[53]
Spent brewery grains	95:5 *v*/*v*; HCl (4 M)	NBT; reflux; 360 min	28.5–32.8	n.r.	n.r.	n.r.	n.r.	n.r.	[54]
Rice husks	95:5–60:40 *v*/*v*; HCl (0.05–0.8 M)	NBT; 5–12 mL/g; 1–16 h min	58.5–78.8	n.r.	n.r.	n.r.	n.r.	n.r.	[50]
Wheat straw	1:1 *v*/*v*; oxalic acid (0–10%)	140–180 °C; 20 mL/g; 30–90 min	n.r.	35.6–64.5	n.r.	n.r.	n.r.	n.r.	[56]
*Eucalyptus* wood	1:1 *v*/*v*; oxalic acid (0–10%)	140–180 °C; 20 mL/g; 60 min	n.r.	56.6–79.4	n.r.	n.r.	n.r.	n.r.	[56]

Nomenclature: n.r., not reported.

### 4.3. PenOH-Based Fractionation

PenOH, a solvent with low water solubility (1.7–2%) and a favorable normal boiling temperature (137 °C) [38,57], has received attention as an agent for LB fractionation in recent years. It has been claimed that the utilization of fractionation media made up of PenOH, water, and an acidic catalyst represents an innovative and promising biorefinery method for the complete valorization of LB, since it allows remarkable energy savings (up to nearly one-third with respect to conventional ethanol fractionation) [37,38], while the solvent can be separated easily via rotary vacuum evaporation [37]. Under conditions compatible with high cellulose recovery yields, this method shows the ability to extensively cleave the ester and ether linkages of lignin, as well as the glycosidic bonds of hemicelluloses [37,38,42,57]. 

Although sulfuric acid is the most used catalyst, recent studies have proposed its replacement by *p*-toluenesulfonic acid, which has advantages in terms of its stability, recyclability (>96%) and low boiling point (140 °C), while enabling efficient solubilization of lignin and hemicellulose under mild pretreatment conditions [58,59]. 

The data summarized in Table 2 confirm that the fractionation effects achieved were largely dependent on the raw material considered and on the operational conditions assayed.

In comparative terms, favorable fractionation of aspen wood and wheat straw was achieved operating under optimal conditions, which led to the extensive removal of lignin and hemicelluloses [37]. Remarkable results (93.4% xylan removal and 89.9% delignification at a 39.9% solid yield) were reported for pine wood (a softwood not very susceptible to other organosolv treatments), but at the cost of increased cellulose loss (32.1%). More selective separation was achieved with wheat straw (93.3% xylan removal with 89.7% delignification and 94.5% cellulose recovery, at a 36.2% solid yield) [57].

The operational conditions were also influential on the lignin properties. When using high water proportions, the aliphatic β-*O*-4 units were absent in the PenOH-soluble lignin fragments [42]. In contrast, operation in media with higher PenOH contents resulted in soluble lignin fractions that can retain a significant part of the native β-*O*-4 linkages. This aspect, together with the low levels of contaminant sugars and high contents of phenolic hydroxyl groups, increase the potential of the PenOH-soluble lignin as a source of biomaterials [37,38,58]. However, harsher treatments may lead to the undesired repolymerization of lignin [32,38].

Concerning the hemicellulose fraction, the hydrolysis reactions decreased with increased PenOH concentrations, a fact ascribed to the lower mobility of hydronium ions (which act as the catalytic species) [38]. High water contents resulted in more than 90% xylan conversion into xylose [42], while higher PenOH concentrations favored the production of xylooligosaccharides (XOS) as the main hemicellulose-derived compounds [57]. This is an interesting finding, since XOS have a remarkable added value, derived from their prebiotic activity, as revealed by the intestinal production of short-chain fatty acids [60] and the modulation of intestinal microbiota by selectively increasing the populations of beneficial bacteria. This fact has been confirmed in fermentation experiments using fecal inocula [61,62,63]. Interestingly, XOS may find specific applications as dietary supplements for the elderly, as their prebiotic effects may counteract the detrimental effects of antibiotics on the gut microflora [64].

### 4.4. MeTHF-Based Fractionation

MeTHF is a polar, aprotic solvent, which has been proposed for LB fractionation owing to a number of favorable features, including [65,66,67,68,69]: its low water solubility; its environmentally friendly character, since its precursors (furfural or levulinic acid) can be directly obtained from LB via hydrolysis–dehydration or hydrolysis–dehydration–rehydration of polysaccharides; its satisfactory stability in acidic environments; its low boiling point (~80 °C), which facilitates its recovery and recycling; its suitability for operation under relatively mild conditions, with no significant generation of undesired byproducts [70]; its easy abiotic degradation by sunlight and air if leaked; and its favorable toxicologic evaluation. 

Scarce information is available on LB fractionation in media containing MeTHF. Although it has been reported to show lower ability to remove lignin than BuOH [56], the efficient lignin separation has been highlighted as an advantage of MeTHF-based processes [71]. In general, the results depend largely on both the raw materials employed and the processing conditions. Specifically, the type of catalyst plays an important role, and controls the major fractionation reactions. For example, strong mineral acids such as sulfuric or phosphoric acids [65,72,73] affect the polysaccharide fraction in a different way to weaker acids [74].

Weak organic acids are the most frequently employed catalysts, although some of the studies carried out with them [70,72,73,75] focus on objectives other than fractionation, providing little information suitable for the purposes of this study. Oxalic acid is the most widely employed catalyst for processing different types of LB, in part due to its less corrosive character than other potential catalysts such as sulfuric acid [67]. Although some studies with oxalic acid provided little useful information on the extent and selectivity of fractionation [56,70], the data listed in Table 3 show a number of general trends. The processing of susceptible substrates, including less lignified feedstocks (straws, bamboo, or perennial biomasses) [67,76,77] and a hardwood [78], resulted in limited lignin removal (71.2% in the best case), with a variation range that does not compare well with the data reported for BuOH or PenOH. In recent studies, oxalic acid has been replaced by 2,5-furandicarboxylic acid (FDCA), a biogenic acid that is claimed to combine economy, efficiency, recoverability, and sustainability [79], and shows higher thermal stability than oxalic acid [80]. However, the data in Table 3 do not show significant improvements when FDCA was used as a catalyst instead of oxalic acid. In a study performed with wheat straw, Zhan et al. [74] considered 20 different kinds of acid catalysts, including organic monobasic acids, organic dibasic acids, organic tribasic acids and sulfuric acid. The best result (up to 77.5% delignification) was achieved with *p*-toluenesulfonic acid (see Table 3), which presented slightly better fractionation ability in comparison with other acidic catalysts, but entailed a significant cellulose loss. Using a different approach, Xue et al. [66] employed diverse Lewis acids (AlCl_3_, CuCl_2_, FeCl_3_ or NiCl_2_) for fractionating birch wood. The best results were obtained with AlCl_3_, which provided delignification percentages similar to other catalysts and enabled the production of solids with more favorable cellulose contents (up to 83.6%), but at the expense of important cellulose loss.

Regarding the properties of the lignin recovered from media composed of MeTHF, water and a catalyst, the results depended on the severity of the operational conditions. Mild treatments provided lignin isolates with low contents of contaminant polysaccharides and structural features typical of native lignins (including abundance of β-*O*-4 linkages), with medium or low molecular weight distributions. These are promising properties for further applications such as the manufacture of value-added aromatic chemicals or renewable polymers [66,67]. However, harsher operational conditions resulted in recondensation and the reduction of β-*O*-4-linkages [80].

As described before for other organosolvents, diverse products can be obtained from hemicelluloses, depending on the operational mode. Using strong mineral acids as catalysts, xylan can be converted into xylose or furfural in one or two reaction steps [65,72,73], while weaker organic acids (oxalic or *p*-toluenesulfonic acids) enabled the xylan conversion into xylose or XOS [74,77].

Simulation and economic assessments [70,71,81] proposed improvements in energy and mass efficiencies through process integration techniques, and concluded that the process could be economically viable, depending on the added value achieved for lignin [71].

## 5. Conclusions

The industrial processes for cellulose pulp manufacture are evolving, looking for higher efficiency, sustainability and profitability. The biorefinery approach (based on the selective fractionation of the raw materials and on the separate utilization of the diverse fractions) provides a valuable conceptual framework. 

The utilization of reaction media containing organic solvents partially soluble in water allows the one-pot fractionation of lignocellulosic substrates: lignin and hemicelluloses are depolymerized (yielding products that appear concentrated in the organic and aqueous phases, respectively), while cellulose is kept in a solid phase. In comparison with conventional organosolv fractionation, the spontaneous separation of lignin- and hemicellulose-derived products increases the energy efficiency and facilitates downstream processing.

The data reported for organosolv fractionation using BuOH, PenOH or MeTHF are scarce and incomplete, but confirm that this operational mode can produce solid substrates with a high cellulose content, together with added-value products derived from hemicelluloses (oligosaccharides, monosaccharides, furans), and high-quality lignin fragments retaining the structure of native lignin. Economic data support the idea that this type of process could be profitable, depending on the added value achieved for the products derived from hemicelluloses and lignin.

## Figures and Tables

**Table 2:** Results reported on the PenOH fractionation of lignocellulosic materials.

Substrate	PenOH:Water Ratio; Catalyst (Conc.)	Temp.; Liquor to Solid Ratio; Time	Solid Yield	Cellulose, %	Klason Lignin, %	Hemic., %	Delignification, %	Cellulose Loss, %	Reference
*Acacia confusa* wood	0–95% *v*/*v*; H_2_SO_4_ (0–0.12 M)	140–200 °C; 5–9 mL/g; 60 min	n.r.	n.r.	n.r.	n.r.	0–70	6–30	[38]
Aspen wood	20–80% *v*/*v*; H_2_SO_4_ (1%)	130–160 °C; 10 mL/g; 60 min	75.3–48.7	55.3–78.5	15.3–3.3	n.r.	44.2–94.6	3.4–13.1	[37]
Monterey pine	150–190 °C; H_2_SO_4_ (1%)	150–190 °C; 10 mL/g; 15–45 min	39.9–90	54.8–95.7	2.8–26.5	n.r.	3.5–89.9	2–22.1	[57]
Wheat straw			30.1–70.7	63.2–96.4	2.3–21.3	n.r.	5.4–89.7	1.3–24.2	
Poplar wood	60:20 *w*/*w*; *p*-toluenesulfonic acid (1 g/g water)	120 °C; 10 mL/g; 40 min	n.r.	86.8	10	n.r.	70		[58]
Aspen wood	60:20 *w*/*w*; *p*-toluenesulfonic acid (1 g/g water)	80–180 °C; mL/g; 40 min	35.4–90.5	68.2–96.4	1.4–14.3	n.r.	33.1–93.3	0.1–17.81	[59]
Moso bamboo	0–98% (*v*/*v*); H_2_SO_4_ (0–8%)	120–140 °C; 6 mL/g; 5–60 min	40.9–94	n.r.	n.r.	n.r.	9–82	0–10	[32]
Sorghum bagasse	12.5% *v*/*v*; H_2_SO_4_ (1%)	180 °C; 13.3 mL/g; 45 min	38	62.2	29	n.r.	74.3	28.1	[42]

Nomenclature: n.r., not reported.

**Table 3:** Results reported on the MeTHF fractionation of lignocellulosic materials.

Substrate	MeTHF:Water Ratio; Catalyst (Conc.)	Temp.; Liquor to Solid Ratio; Time	Solid Yield	Cellulose, %	Klason Lignin, %	Hemicellulose, %	Delignification, %	Cellulose Loss, %	Reference
Wheat straw	1:1 *v*/*v*; oxalic acid (1–7 wt%)	140–160 °C; 20 g/L; 60 min	n.r.	35.1–56.6	n.r.	n.r.	n.r.	n.r.	[56]
Bamboo	1:1 *v*/*v*; oxalic acid (1 M)	140–180 °C; 10 mL/g; 20 min	41.9–74.3	58.5–72.5	26–28	0–19	23.9–56.4	7–35	[67]
Beech wood	1:1 *v*/*v*; oxalic acid (1 M)	85–150 °C; 80–140 °C; 20 mL/g; 180 min	50–84	n.r.	n.r.	n.r.	60–70	n.r.	[78]
Rice straw	1:1 *v*/*v*; oxalic acid (0.1 M)	125–160 °C; 12.5 mL/g; 25–45 min	48.75–69.5	47.85–62.5	5.57–14.85	4.74–16.69	16.4–71.3	1–42.6	[76]
Perennial plant biomasses	1:1 *v*/*v*; oxalic acid (0.1 M)	140 °C; 20 mL/g; 180 min	40–54	46–63	15–18	n.r.	~60–70		[77]
Beech wood	FDCA (0.1 M)	140–160 °C; 20 mL/g; 60–180 min	n.r.	n.r.	n.r.	n.r.	30.7–66.7	n.r.	[80]
Wheat straw	1:1 *v*/*v*; *p*-toluenesulfonic acid (0.05–1 M)	100–180 °C; 20 mL/g; 90–360 min	n.r.	n.r.	n.r.	n.r.	29.9–77.5	0–26.7	[74]
Birch wood	1:1 *v*/*v*; AlCl_3_ or CuCl_2_ or FeCl_3_ or NiCl_2_ (0.1 M)	140–180 °C; 20 mL/g; 60 min	42–70	60–83.6	13.427	0–10	45–74.8	7.3–59.2	[66]

Nomenclature: n.r., not reported.

## Data Availability

The data are available on request.
